# HMGB1 in Septic Muscle Atrophy: Roles and Therapeutic Potential for Muscle Atrophy and Regeneration

**DOI:** 10.1002/jcsm.13711

**Published:** 2025-02-18

**Authors:** Si‐Yuan Qi, Qiqi Wu, Peng‐Hui Xiang, Chao‐Yao Hou, Zhaofeng Kang, Meng‐Qi Chen, Chengla Yi, Xiangjun Bai, Tianyu Li, Zhanfei Li, Wei‐Ming Xie

**Affiliations:** ^1^ Division of Trauma Surgery, Emergency Surgery & Surgical Critical, Tongji Trauma Center, Tongji Hospital, Tongji Medical College Huazhong University of Science and Technology Wuhan China; ^2^ Department of Emergency and Critical Care Medicine, Tongji Hospital, Tongji Medical College Huazhong University of Science and Technology Wuhan China; ^3^ Department of Respiratory and Critical Care Medicine, Tongji Hospital, Tongji Medical College Huazhong University of Science and Technology Wuhan Hubei China; ^4^ Trauma Center Peking University People's Hospital Beijing China; ^5^ Key Laboratory of Trauma Treatment and Neural Regeneration (Peking University) Ministry of Education Beijing China; ^6^ National Center for Trauma Medicine of China Beijing China

**Keywords:** clinical therapy, HMGB1, muscle atrophy, sepsis, tissue regeneration

## Abstract

Currently, the treatment of septic myopathy presents significant challenges with implications for increased mortality rates and prolonged hospitalizations. Effective therapeutic strategies for septic myopathy remain elusive, highlighting an urgent need for novel therapeutic approaches. High‐mobility group box 1 (HMGB1) is a conserved nonhistone nuclear protein that is released passively from deceased cells or actively secreted by activated immune cells, influencing both infectious and noninfectious inflammatory responses. Studies have indicated that HMGB1 likely plays a pivotal role in the pathogenesis of septic myopathy by crucial pathways associated with muscle atrophy and contributing to muscle regeneration under certain conditions. This review aims to summarize the possible mechanisms of HMGB1 in muscle atrophy and its potential in muscle regeneration, providing a theoretical basis for HMGB1 treatment of septic myopathy. Research shows that the dual role of HMGB1 is related to its specific forms, which are influenced to varying degrees by environmental factors. HMGB1 is a key participant in septic muscle atrophy, whereas HMGB1 shows therapeutic potential in muscle regeneration. One key mechanism by which HMGB1 contributes to septic muscle atrophy is through the exacerbation of inflammation. HMGB1 can amplify the inflammatory response by promoting the release of pro‐inflammatory cytokines, which further damages muscle tissue. HMGB1 is also involved in promoting cell death in sepsis, which contributes to muscle degradation. Another important mechanism is the regulation of protein degradation systems. HMGB1 can activate the ubiquitin–proteasome system and autophagy–lysosome pathway, both of which are crucial for the breakdown of muscle proteins during atrophy. Conversely, targeting HMGB1 has shown the potential to ameliorate muscle atrophy in various diseases. For instance, HMGB1 has been shown to promote muscle vascular regeneration, modify stem cell status and enhance stem cell migration and differentiation, all of which are beneficial for muscle repair and recovery. Pharmacological inhibition of HMGB1 has been explored, with several drugs demonstrating efficacy in reducing inflammation and muscle degradation in sepsis models. These findings suggest that HMGB1 inhibition could be a viable therapeutic approach for septic myopathy. However, the function of promoting muscle regeneration in septic myopathy needs further research. HMGB1 emerges as a promising therapeutic target for the treatment of muscle atrophy in sepsis. This review focuses on identifying the correlation between HMGB1 and septic myopathy, analysing the possible role of HMGB1 in disease development and examining the feasibility of HMGB1 as a therapeutic target.

## 1 Introduction

The primary function of skeletal muscles is to maintain vital activities such as posture, locomotion and respiration through mechanical movements, which depend on the contraction of muscle fibres. Thermogenesis and participation in the maintenance of metabolic homeostasis play their principal roles [1]. Skeletal muscles contribute significantly to thermogenesis and metabolic homeostasis. Muscle mass maintenance depends on the delicate balance between protein synthesis and degradation, which is influenced by nutritional status, hormonal equilibrium, physical activity, trauma and disease conditions [2]. Muscle atrophy is the loss of muscle mass and strength [3]. Features of skeletal muscle atrophy include loss in muscle fibres or shrinkage in diameter—the most significant part, changes in fibre type or myosin isoforms and a net loss of cytoplasm, organelles and total protein [1]. These pathological changes are prevalent in patients with septic muscle atrophy. Patients with sepsis tend to suffer from many issues owing to their intense, ongoing inflammation. Muscle atrophy is a distinctive feature that causes many adverse effects on the patient, affecting the quality of life, prolonging recovery from the disease, leading to extended hospital stays and rendering patients susceptible to the development of secondary diseases.

As a late‐phase inflammatory mediator, HMGB1 plays a crucial role in sepsis pathophysiology. The release of HMGB1 is closely associated with the inflammatory response, organ dysfunction and mortality in patients with sepsis. By inhibiting the release of HMGB1 or blocking its pathways, the systemic inflammatory response induced by sepsis can be effectively mitigated, thereby improving patient outcomes. HMGB1 plays a crucial role in the development of muscle atrophy during sepsis via multiple mechanisms.

Currently, treatment strategies for muscle atrophy, embracing physical exercise, nutritional support, drug therapy and other methods, exhibit limited efficacy. The role of HMGB1 in muscle atrophy remains controversial. In septic myopathy, it can both exacerbate muscle wasting and, under certain circumstances, facilitate muscle regeneration. This duality underscores the complexity and contradictions in HMGB1's biological functions. The investigation of septic myopathy provides a pertinent framework for exploring the role of HMGB1 and its therapeutic potential in managing muscle atrophy. In this review, we examine the diverse roles of HMGB1 in various states, emphasizing its potential mechanisms for inducing inflammation and muscle atrophy. We investigated the effect of HMGB1 on tissue regeneration under specific conditions. These insights into the multifaceted functions of HMGB1 provide a comprehensive understanding of its influence on both pathological and regenerative processes.

## 2 The Structures, Modifications and Functions of HMGB1

### 2.1 The Structures and Modifications of HMGB1

HMGB1, an evolutionarily highly conserved nonhistone chromatin‐binding protein, belongs to a subfamily of the HMG proteins and is present in nearly all cell types. HMGB1 is typically primarily found in the nucleus, where it can promote the assembly of nucleosomes, distort DNA and play a role in transcription, replication and DNA repair. In pathological conditions, however, HMGB1 undergoes posttranslational and redox modifications that can affect its distribution and subsequent function [4]. Specifically, HMGB1 expression may increase under stress or injury, during which posttranslational modifications of HMGB1 involve acetylation, phosphorylation, ADP‐ribosylation, methylation and glycosylation, which affect its localization and function, highlighting the complex regulatory role of HMGB1 in cellular physiology and pathology [5].

**FIGURE 1:**
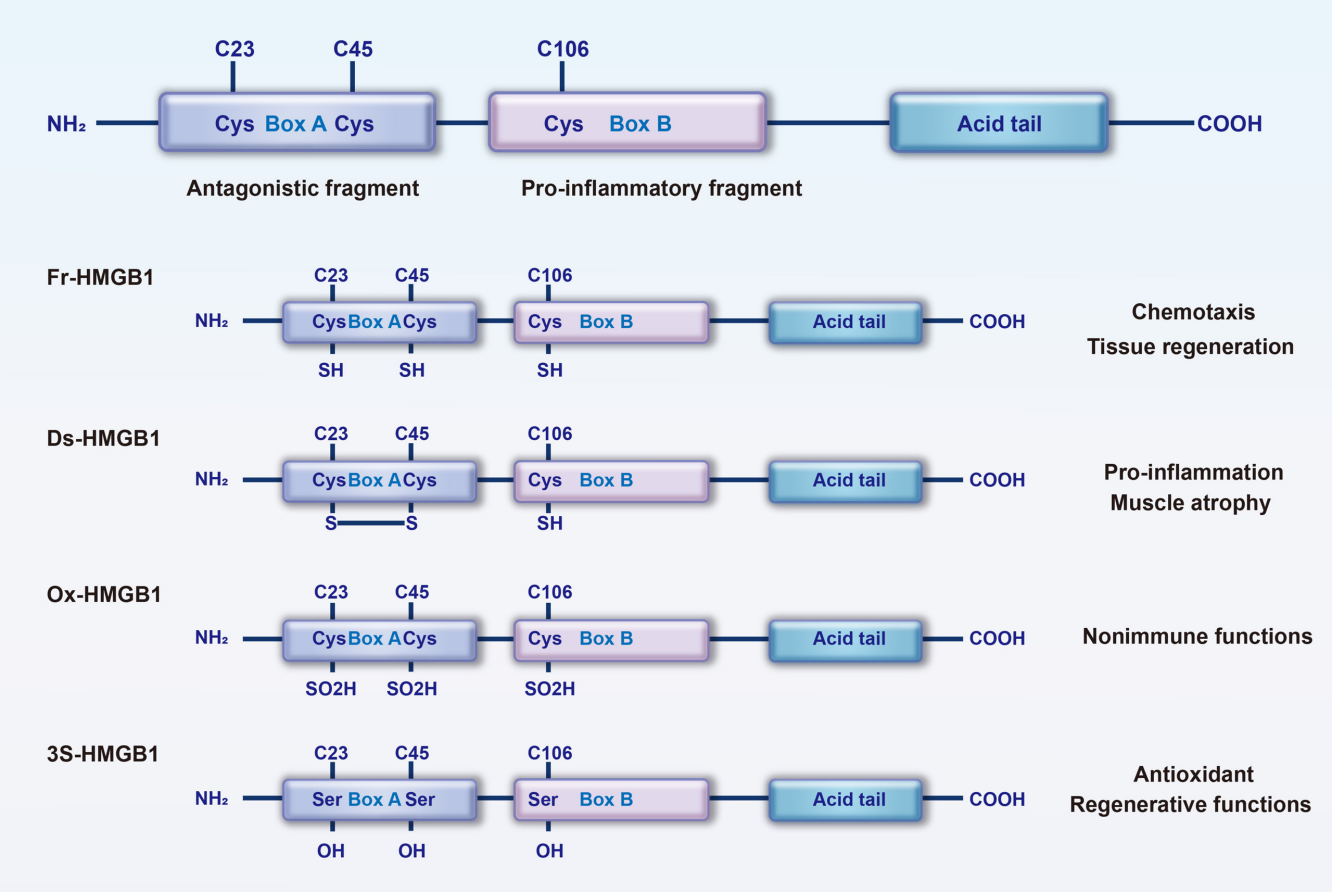
HMGB1 structure and function. HMGB1 has three main structural domains: Box A, Box B and the acidic tail. Box A and Box B exhibit opposing functions, with Box A acting antagonistically and Box B promoting inflammation. HMGB1 exists in three forms, each associated with different functions. Fully reduced HMGB1 (Fr‐HMGB1) primarily exhibits chemotactic properties, disulfide HMGB1 (Ds‐HMGB1) mainly has pro‐inflammatory effects, and oxidized HMGB1(Ox‐HMGB1) lacks immunological activity. Triple serine‐mutated HMGB1 (3S‐HMGB1) has antioxidant properties and achieved better muscle and liver regeneration than endogenous HMGB1.

Its three cysteine residues (Cys23, Cys45 and Cys106) located in Box A and Box B, respectively, are susceptible to redox modification, allowing it to exist in three main forms, each with a functionally distinct function [6] (Figure 1). The corresponding three main forms are fully reduced HMGB1 (Fr‐HMGB1), disulfide HMGB1 (Ds‐HMGB1) and oxidized HMGB1 (Ox‐HMGB1). In Fr‐HMGB1, the cysteine residues are unoxidized, preventing the mediation of inflammatory reactions, although they can still bind to receptors for chemotaxis [7, 8, 9, 10]. Fr‐HMGB1 has chemotactic effects and is associated with stem cell regeneration. It does not polarize macrophages towards M1 or M2 phenotypes but does induce cell migration [11, 12]. The Ds‐HMGB1, which forms disulfide bonds between 23 and 45 and retains the reduced 106cys, can stimulate the secretion of cytokines or chemokines by binding to Toll‐like receptor (TLR)4 or binding to Receptor for Advanced Glycation Endproducts (RAGE) [7, 10]. Ds‐HMGB1 serves as a biomarker of inflammation and a therapeutic target for inhibiting inflammatory responses. Ds‐HMGB1 is highly expressed in normal spleen and liver tissues but low in muscle, with elevated levels observed after acute muscle injury [13]. Ox‐HMGB1, with all three cysteine residues oxidized, could not play the role of either inducing inflammation or recruiting stem cells as a Chemoattractant [6, 8, 10]. Significantly, substituting these cysteine residues with serine in the Triple Serine‐mutated HMGB1 (3S‐HMGB1) renders it resistant to environmental redox changes. It was found that 3S‐HMGB1 has antioxidant properties and achieved better muscle and liver regeneration than endogenous HMGB1 [10]. This was attributed to the fact that it does not undergo oxidation, preserving its chemotactic and regenerative effects and not being converted to a pro‐inflammatory form.

### 2.2 The Function of HMGB1

In response to microbial invasion or noninfectious injury, HMGB1 is released into the extracellular space, where it actively participates in inflammatory processes. As a significant mediator of both acute and chronic inflammation, HMGB1 is implicated in the onset and progression of numerous conditions, including systemic lupus erythematosus, myositis, sepsis, trauma, respiratory disorders, myocardial infarction, atherosclerosis, cancer and cerebral diseases [14, 15, 16]. In sepsis, HMGB1 levels are significantly elevated, and its release occurs later and persists longer than other inflammatory mediators, identifying it as a ‘late’ inflammatory mediator. Through downstream signalling pathways, HMGB1 induces the production of numerous inflammatory factors, leading to an uncontrolled inflammatory response and worsening tissue damage. The link between HMGB1 and sepsis is also reflected in the close association with septic organ damage. HMGB1 serves as a critical mediator of organ damage, associated with inflammation and dysfunction in organs such as the lung, brain, liver, kidney and heart [17, 18, 19, 20] (Figure S1). Its role in promoting inflammatory responses and tissue injury highlights its impact on sepsis‐related complications. In patients with sepsis, muscle atrophy is commonly observed. HMGB1 is also closely associated with the progression of this septic muscle atrophy. It promotes various forms of cell death, triggers inflammation, worsening muscle loss. HMGB1 also drives muscle protein degradation through the ubiquitin–proteasome system and autophagy–lysosome pathways.

HMGB1 is of vital significance to embryogenesis, muscle development and growth. HMGB1 deficiency in mice results in perinatal death and reduced muscle mass, underscoring its role in muscle formation [21]. HMGB1 has been identified as a contributing factor in promoting several regenerative processes, such as in skin wound healing models, models of myocardial and peripheral tissue ischemia and vascular endothelial growth factor (VEGF)–dependent angiogenesis in diabetic models [22]. In vivo studies have shown that HMGB1 promotes regeneration in muscle, bone and haematopoietic tissues [23]. During muscle injury or inflammation, whereas HMGB1 has pro‐inflammatory effects, it is also critical for muscle regeneration. Based on Bianchi et al. [24], we believe there are several basic explanations for this. At the site of tissue injury, HMGB1 attracts macrophages. During the inflammatory process, these macrophages transition from an inflammatory to a tissue‐healing phenotype. HMGB1 also attracts both local and distant stem cells, promoting their differentiation into muscle cells to support muscle regeneration and repair. HMGB1 can alter cells to pre‐proliferative, enabling more rapid and effective regeneration after injury. Also, HMGB1 promotes angiogenesis, which could further aid in muscle regeneration.

**FIGURE 2:**
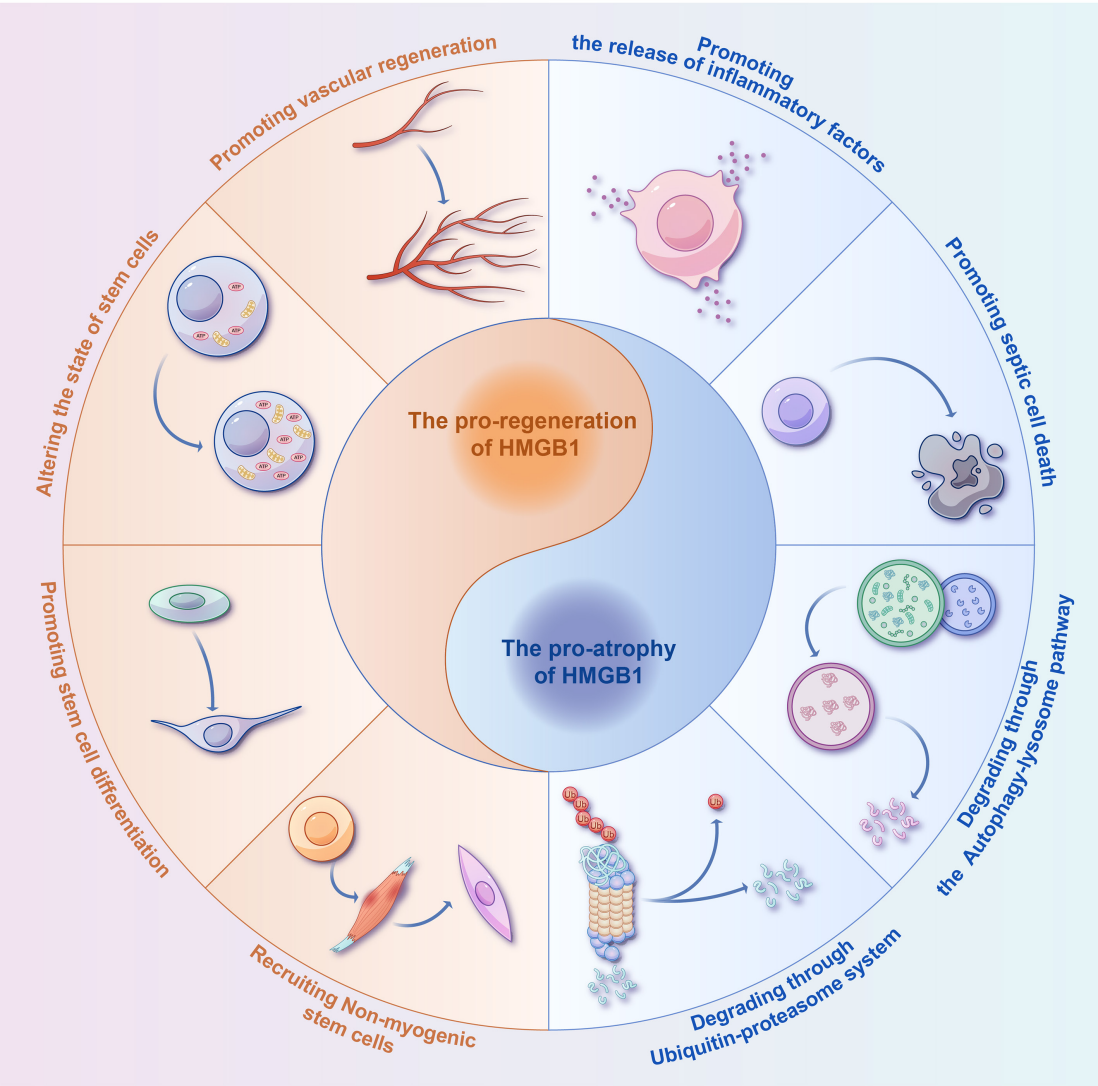
HMGB1 in muscle regeneration and atrophy. Pro‐atrophic actions of HMGB1: 1. Induces release of inflammatory cytokines: HMGB1 triggers the release of pro‐inflammatory cytokines, contributing to tissue degradation; 2. Promotion of cell death: It enhances cell death, leading to tissue atrophy; 3. Protein degradation via autophagy–lysosome pathway: HMGB1 stimulates the autophagy–lysosome pathway to degrade cellular proteins; 4. Protein degradation via ubiquitin–proteasome system: It also promotes protein degradation through the ubiquitin–proteasome system, contributing to muscle wasting. Pro‐regenerative actions of HMGB1: 1. Promotes angiogenesis in muscles: HMGB1 facilitates the formation of new blood vessels within muscle tissue; 2. Modulates stem cell states: It alters the state of stem cells, enhancing their ability to support tissue repair; 3. Enhances stem cell differentiation: HMGB1 promotes the differentiation of stem cells into specialized cell types necessary for tissue regeneration; 4. Recruits mesenchymal stem cells (MSCs): It attracts MSCs to the site of injury, aiding in the repair and regeneration processes.

Therefore, HMGB1 has a dual role in muscle atrophy. During sepsis, it contributes to muscle wasting through various pathways and also plays a crucial role in muscle regeneration through multiple mechanisms (Figure 2). We further elaborate on its two‐sided nature below.

## 3 Mechanisms of HMGB1‐Induced Muscle Atrophy

Sepsis is associated with catabolism in skeletal muscle, particularly the breakdown of myofibrillar proteins such as myosin [25]. It can significantly decrease protein synthesis in skeletal muscle [25]. Excessive loss of muscle mass is a poor prognostic indicator of the disease, which may be as minor as affecting the quality of life of patients or as significant as aggravating the progression of the disease and even endangering life. Existing in septic muscle, the two major pathways, ubiquitin protease and autophagy–lysosome pathways, regulate protein degradation and muscle mass. In parallel, there are several other pathways that affect muscle protein degradation including the calpain‐dependent pathway and the caspase‐dependent pathway.

**FIGURE 3:**
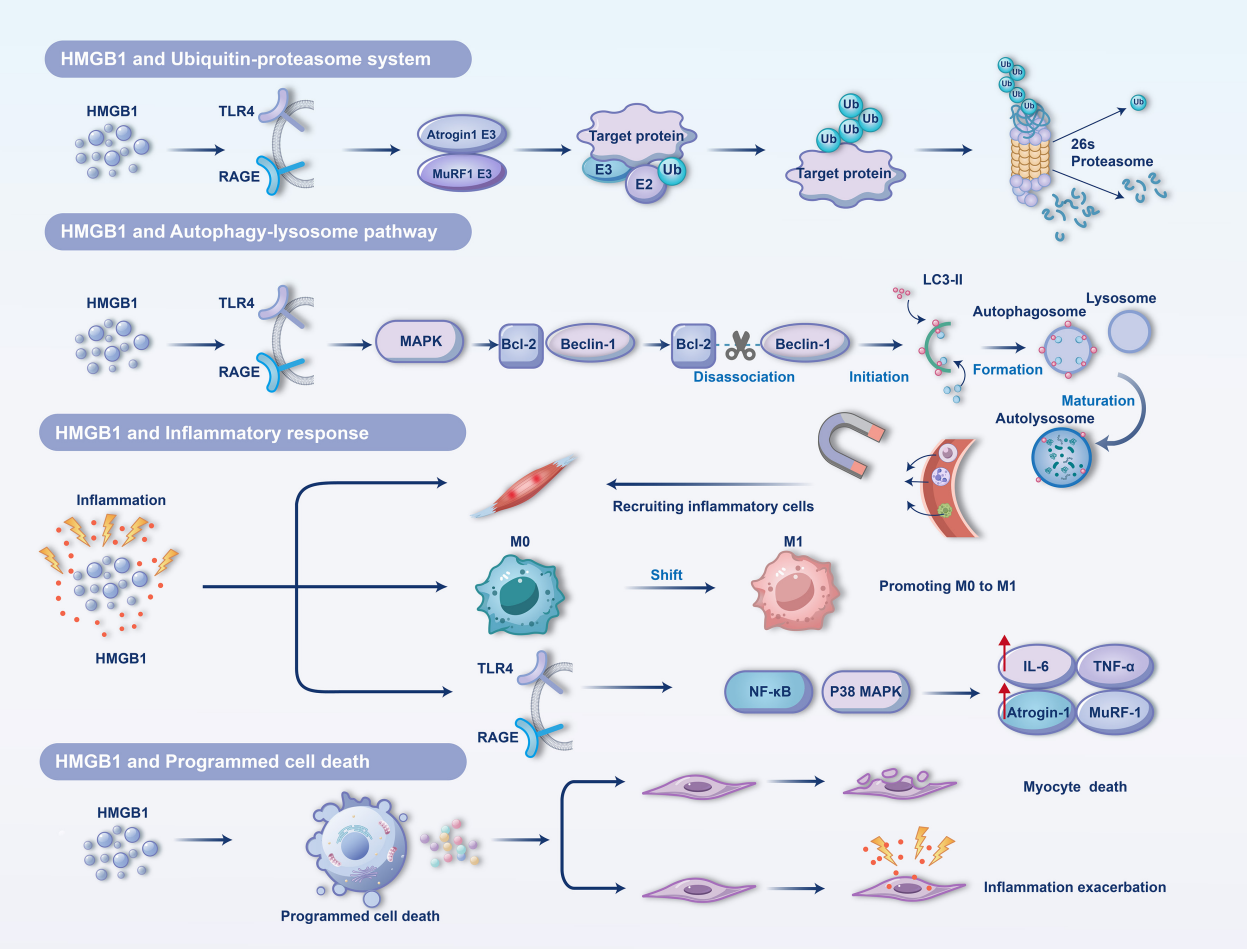
Mechanisms by which HMGB1 affects muscle atrophy. 1. HMGB1 and ubiquitin–proteasome system: HMGB1 activates the expression of two E3 ligases, Atrogin1 and MuRF1, the downstream muscle atrophy protein genes, through TLR4 and RAGE, which sequentially ubiquitinates the target proteins for tagging and then undergoes proteasomal degradation. 2.HMGB1 and autophagy‐lysosome pathway: HMGB1 activates downstream MAPK genes through TLR4 and RAGE, which promotes the dissociation of BCL‐2 and Beclin1, contributing to the elevation of LC3‐II, which in turn initiates the autophagy process, sequentially forming autophagic lysosomes and then degrading the target protein. 3. HMGB1 and Inflammatory response: HMGB1 is mainly involved in muscle atrophy in inflammation by a. recruiting inflammatory cells to reach damaged muscles, b. prompting the transition of macrophage M0 to M1 and c. prompting the expression of inflammatory factors and atrophic genes through downstream signalling pathways. 4. HMGB1 and programmed cell death: HMGB1 is involved in a multitude of programmed cell deaths in sepsis, which in turn can directly trigger myocyte death or exacerbate myocyte inflammation, with consequent effects on muscle atrophy.

Inflammation and programmed cell death also play crucial roles in muscle atrophy associated with sepsis. Sepsis commonly triggers a systemic inflammatory response, resulting in the excessive release of pro‐inflammatory factors. These factors contribute to muscle atrophy by activating downstream signalling pathways that enhance protein degradation while suppressing protein synthesis. Furthermore, pro‐inflammatory factors can induce programmed cell death in muscle cells, compromising cell integrity and causing a decline in muscle mass. Programmed cell death not only directly leads to muscle loss but also intensifies the local inflammatory response, establishing a vicious cycle that accelerates muscle atrophy progression. The four main HMGB1‐related mechanisms discussed here are depicted in Figure 3.

### 3.1 HMGB1 and Ubiquitination–Proteasome System

Within the ubiquitin–proteasome system, proteins are tagged with a polyubiquitin chain for degradation by the 26S proteasome, which comprises two subunits, 19S and 20S. This tagging mechanism primarily involves three categories of ligases: E1, E2 and E3. These ligases facilitate protein ubiquitination through covalent attachment [26]. Upregulation of this pathway is a common denominator of various causes of muscle atrophy. Of these, E3 ligases primarily recognize the target, assemble the complex with the E2 enzyme and catalyse the transfer of ubiquitin from E2 to the substrate, which represents the rate‐limiting step of the ubiquitination process that regulates subsequent proteasome‐dependent degradation [27]. The two critical muscle‐specific Atrogin1 (also known as MAFbx) and muscle‐specific RING finger protein 1 (MuRF‐1) are the most intensively researched of the E3 ubiquitin ligases to date and are implicated in almost all forms of muscle atrophy [3]. The expression of these two ubiquitin ligases was significantly increased in a rat model of skeletal muscle atrophy in sepsis [28]. Chung et al. identified HMGB1 as an upstream regulator of Atrogin1 expression and that this signalling pathway activates Atrogin1 via downstream nuclear factor kappa–light chain enhancer of activated B cells (NF‐κB) to ubiquitously tag aquaporin 4 for degradation, resulting in shrinkage of muscle cell counts and types [29]. It was found that HMGB1 can function on TLR4 and NF‐κB to promote the expression of muscle atrophy markers Atrogin1 and MuRF‐1. Treatment with TAK‐242, a TLR4 inhibitor, as well as glycyrrhizin and BAY 11‐7082, inhibitors of HMGB1 and NF‐κB, respectively, resulted in the inhibition of Atrogin1 and MuRF‐1 expression and mitigated muscle atrophy [30]. Deficiency in myostatin resulted in withdrawn expression in HMGB1 and drawback in the expression of the atrophy‐related genes MuRF‐1 and Atrogin1, accompanied by inhibited activation of the Janus kinase (JAK) signal transducer and activator of transcription protein 3 (STAT3) pathway to reduce muscle atrophy in septic mice [31].

### 3.2 HMGB1 and Autophagy–Lysosome Pathway

Autophagy is a conserved process in eukaryotic cells that relies on the lysosomal pathway for the degradation of cytoplasmic proteins and organelles, which is comparable to an intracellular scavenger utilizing degradation products for recirculation, contributing to cellular renewal and homeostasis [32]. Autophagy maintains cellular homeostasis against sterile and infectious stimuli and cell survival by degrading unnecessary processes for the necessary ones. However, defective or excessive autophagy can lead to various diseases and pathological conditions [33].

HMGB1 plays a crucial role in promoting autophagy and regulating apoptosis. HMGB1 can replace B‐cell lymphoma 2 (BCL‐2) in binding with Beclin1, thereby promoting the activation of autophagy [34]. Studies have revealed that HMGB1 deficiency results in reduced accumulation of autophagy‐associated Microtubule‐associated protein 1A/1B–light chain 3 (LC3) puncta, decreased expression of LC3‐II, elevated levels of Sequestosome 1 (SQSTM1/ p62 protein) and impaired autophagosome formation [35]. Further investigations have confirmed that TLR4 can induce autophagy through downstream signalling involving receptor‐interacting protein kinase 1 (RIPK1) and p38 mitogen‐activated protein kinase (MAPK) [36]. HMGB1 triggers inflammatory responses by binding to TLR4, subsequently promoting the production of inflammatory cytokines and activating autophagy. This activation further exacerbates inflammatory responses mediated by TLR4 signalling during lung ischemia/reperfusion injury [37]. Notably, lipopolysaccharide (LPS) activation of TLR4/p38 MAPK induces C2C12 myotube atrophy by upregulating autophagosome formation and expression of Atrogin1 and MuRF‐1 [38]. These suggest its potential regulation of autophagy through TLR4 in muscle atrophy.

HMGB1 activates extracellular signal‐regulated kinase (ERK) by binding to RAGE, which further activates death‐associated protein kinase (DAPK) and promotes the phosphorylation of Beclin‐1 and dissociation from Bcl‐2, thereby regulating autophagy [39]. In colon cancer, HMGB1 released by cancer cells induces p38 phosphorylation via RAGE, promoting autophagy. This leads to muscle protein degradation, thereby providing energy for the cancer cells [40]. Another study highlights the similar role of HMGB1/autophagy in denervated muscle atrophy [41].

### 3.3 HMGB1 and Inflammatory Response

Sepsis is commonly associated with a pronounced systemic inflammatory response. While inflammation is crucial for immune defence, wound healing and regeneration, persistent unresolved inflammation can detrimentally impact tissue repair and regeneration, potentially leading to chronic conditions like fibrosis and muscle atrophy, particularly when the underlying injurious factors persist [42]. HMGB1 released by injured cells can activate immune cells, producing and releasing inflammatory factors. Inflammatory cytokines such as tumour necrosis factor–alpha (TNF‐α), interleukin‐1 beta (IL‐1β) and interleukin‐6 (IL‐6) are pivotal in the pathogenesis of muscle atrophy. These cytokines can promote muscle protein degradation and inhibit protein synthesis, culminating in muscle wasting and functional impairment [43].

Inflammation contributes to muscle atrophy in septic mice and patients with chronic kidney disease through the activation of TLR4, which triggers NF‐κB and p38 MAPK signalling pathways [44, 45]. In Duchenne muscular dystrophy, HMGB1 plays a critical role in muscle atrophy through inflammation. Genetic deletion of TLR4 in affected mice improved muscle strength, reduced fibrosis and shifted macrophage polarization towards an anti‐inflammatory state. Glycyrrhizin treatment further reduced inflammation and enhanced muscle function [46]. RAGE, activated by elevated HMGB1 and S100 calcium‐binding protein B (S100B), promotes catabolic pathways via p38MAPK, contributing to muscle degeneration [47]. Persistent HMGB1 release from macrophages exacerbates inflammation, whereas RAGE inhibition alleviates the immune response and reduces muscle damage [48]. Inflammatory factors can also promote muscle atrophy by up‐regulating the expression of components of the ubiquitin–proteasome system [30].

Inflammation upregulates HMGB1 and affects its status and function, and reciprocally, HMGB1 contributes to inflammation. HMGB1 exerts recruitment to attract inflammatory cells in inflammation. Ds‐HMGB1 is closely linked to inflammation and is considered both a biomarker and a therapeutic target [13]. Fr‐HMGB1 promotes stem cell recruitment for tissue repair and can induce macrophage migration, but does not polarize macrophages towards the M1 or M2 phenotype, whereas Ds‐HMGB1 polarizes macrophages towards the pro‐inflammatory M1 phenotype [11, 12]. Compared to endogenous HMGB1, 3S‐HMGB1 shows superior regenerative effects, likely due to reduced inflammation [10]. When the oxidized Box A is fully reduced by dithiothreitol (DTT), HMGB1's redox state can shift from a pro‐inflammatory to a regenerative state [49, 50]. This change in HMGB1 status influences the activity of macrophages and stem cells in skeletal muscle inflammation [51]. Modifying HMGB1's redox state can stimulate immune responses or induce immune tolerance in specific situations [52]. Understanding the mechanisms behind HMGB1 oxidation offers new strategies for controlling immune responses and promoting tissue regeneration.

### 3.4 HMGB1 and Programmed Cell Death

**FIGURE 4:**
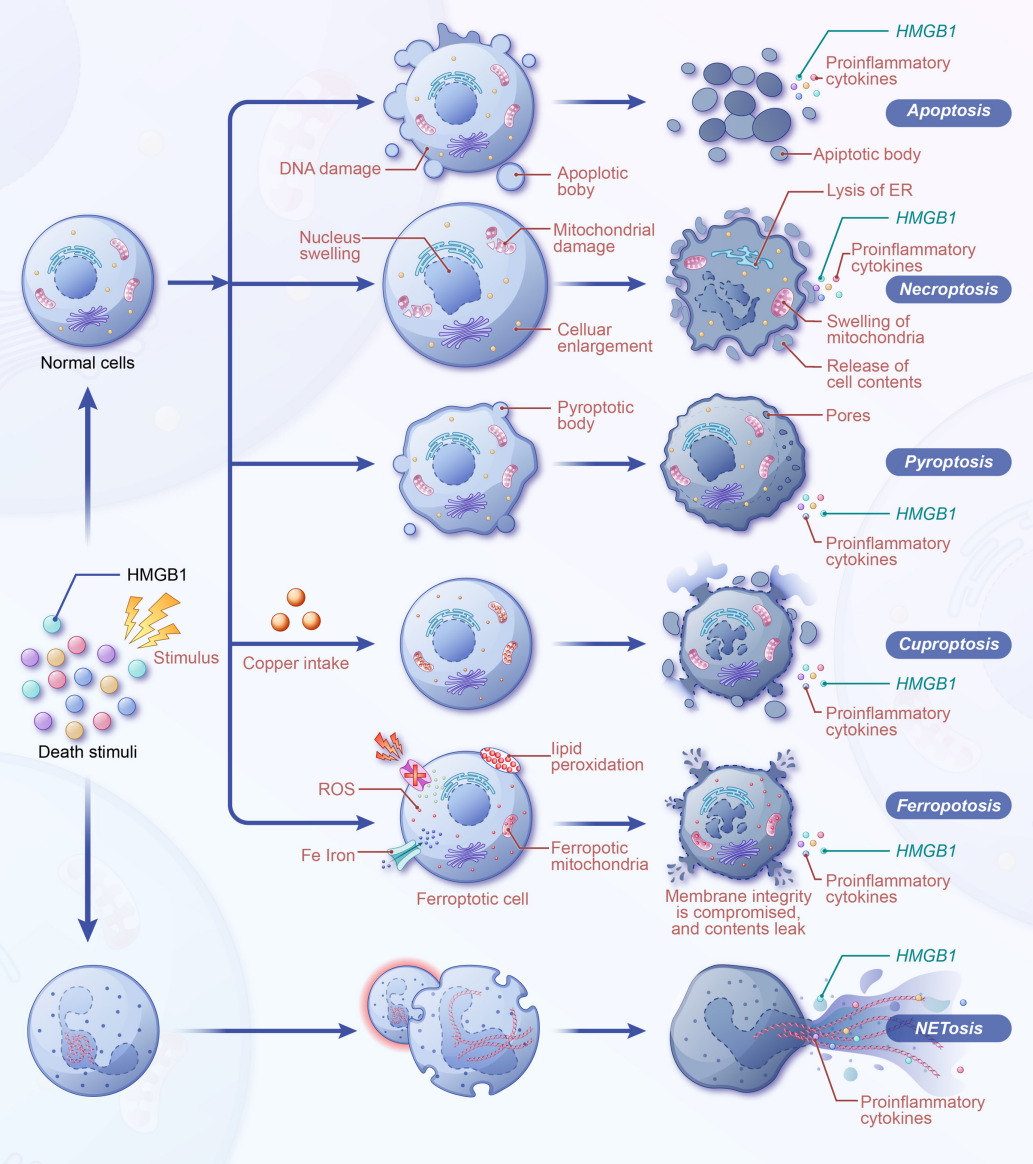
HMGB1 in programmed cell death during septic muscle atrophy. HMGB1 is implicated in multiple forms of cell death during sepsis and continues to be released post–cell death, thereby intensifying inflammation, inducing further cellular demise and contributing to muscle atrophy.

HMGB1 is closely associated with various modes of cell death in sepsis, and its role in skeletal muscle atrophy is significant (Figure 4). Following programmed cell death, HMGB1 is released as an inflammatory signalling molecule, exacerbating immune and inflammatory responses. This release not only promotes further cellular damage but also has a direct impact on muscle cells, potentially leading to their death.

Passive release of HMGB1 has been observed in apoptotic cells [53]. In certain cases, HMGB1 may be involved in the regulation of apoptosis. During pyroptosis, double‐stranded RNA‐dependent protein kinase (PKR) induces inflammasome formation, activates caspase‐1 and subsequently promotes HMGB1 release [54]. In sepsis, HMGB1 is involved in a series of cascade reactions that induce cellular pyroptosis, promoting the inflammatory process [55]. In inflammatory diseases such as sepsis, cells undergoing necroptosis also release large amounts of HMGB1, which further participates in the inflammatory response [56]. Correspondingly, HMGB1‐related pathways can also induce necroptosis [57]. Autophagy‐mediated inhibition of histone deacetylase promotes HMGB1 acetylation, leading to HMGB1 release during ferroptosis [58]. The HMGB1 pathway induces ferroptosis in sepsis‐related diseases, resulting in inflammatory responses and organ damage [59]. Studies have shown that cuproptotic cells can also release HMGB1 to trigger inflammation. Adenosine triphosphate (ATP) depletion due to copper accumulation activates AMP‐activated protein kinase (AMPK), promoting HMGB1 phosphorylation and increasing extracellular release [60]. Some studies have suggested a potential link between cuproptosis and sepsis [61]. Studies have revealed that the induction of neutrophil extracellular trap formation (NETosis), platelet activation, NETosis propagation and subsequent thrombosis heavily depend on extracellular HMGB1 [62]. NETosis is also closely linked to the development of sepsis [63].

The connection between HMGB1 and multiple forms of cell death underscores its role in the inflammatory cascade. For instance, HMGB1 is released from necrotic cells and can also be involved in apoptosis and pyroptosis. The release of HMGB1 intensifies inflammation and promotes cellular damage, creating a detrimental cycle that may exacerbate muscle atrophy and directly lead to programmed cell death in muscle cells.

## 4 Utilizing HMGB1 for Muscle Regeneration

HMGB1 also plays a critical role in muscle development and regeneration. HMGB1−/− mice exhibit reduced muscle mass, underscoring the importance of HMGB1 in muscle development [21]. According to Dormoy et al., maintenance of high HMGB1 expression is essential for embryonic myogenesis and muscle regeneration following injury [64]. Studies have demonstrated that HMGB1 regulates the function of myogenic myoblasts and endothelial cells (ECs) to mediate skeletal muscle regeneration [65]. Human antigen R (HuR) is significantly central to the early stages of myogenesis and is correlated with the expression of HMGB1 in muscle cells [64]. In muscle cells, miRNA‐1192 straightly suppressed the translation of HMGB1, whereas HuR promotes myogenesis by myoblasts by enhancing HMGB1 translation and inhibiting miR‐1192‐mediated translational repression [64]. Depletion of HMGB1 significantly decreased the efficacy of muscle fibre production, the levels of myosin heavy chain (MyHC) and the degree of myoglobin. Exposure of endogenous HMGB1‐depleting C2C12 cells to recombinant HMGB1 restored their capacity to engage in the myogenic stage [64].

**FIGURE 5:**
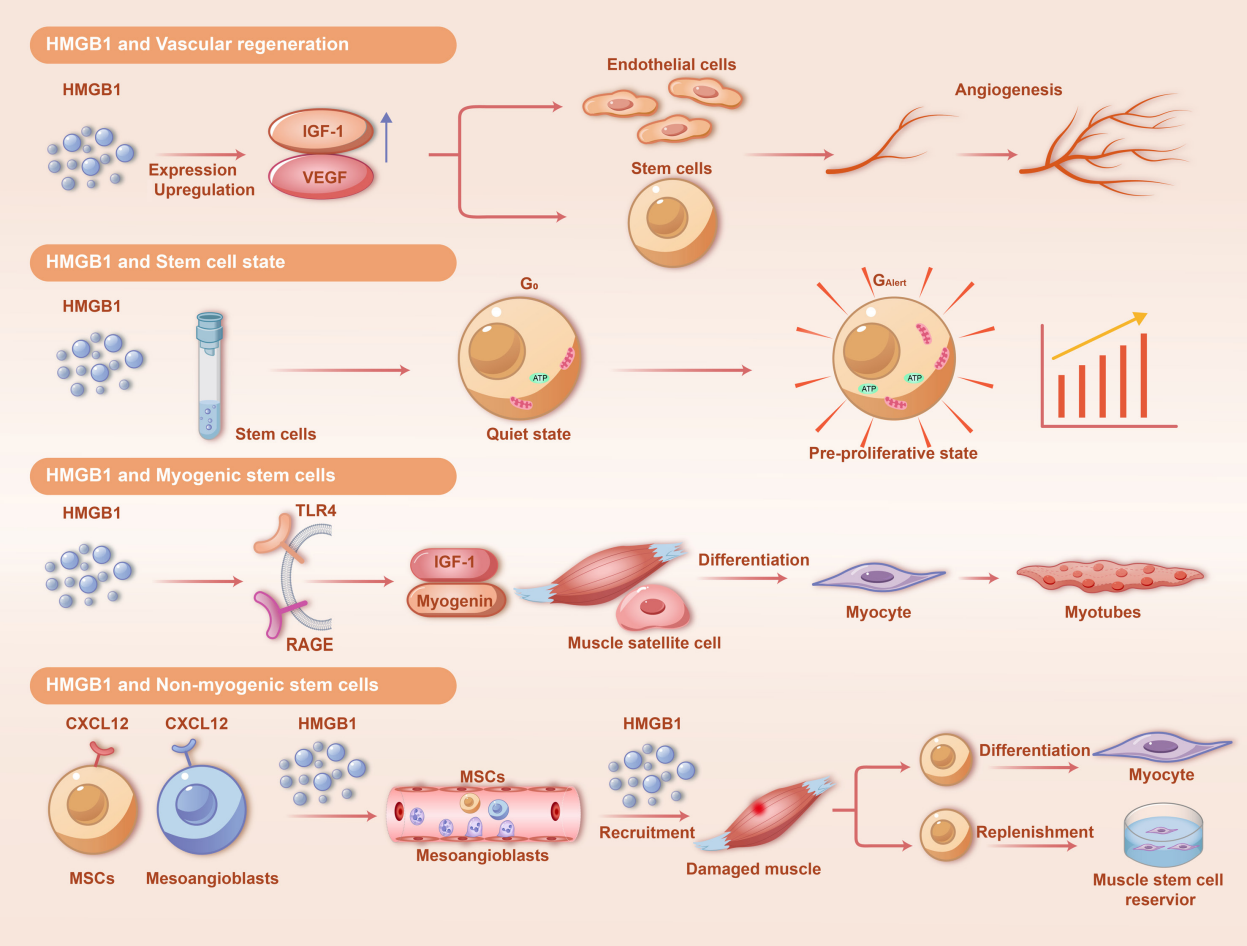
Mechanisms by which HMGB1 promotes muscle regeneration. 1. HMGB1 and vascular regeneration: HMGB1 upregulates the expression of IGF‐1 and VEGF, which in turn promotes the differentiation of ECs and stem cells for angiogenesis. 2. HMGB1 and stem cell state: Alterations in resting stem cells can be promoted by administration of HMGB1, as reflected in increased proliferative potential to better cope with alterations such as muscle damage. 3. HMGB1 and myogenic stem cells: HMGB1 activates the expression of IGF‐1 and myogenin in muscle satellite cells through the downstream pathways, prompting the differentiation of satellite cells and muscle production. 4. HMGB1 and non‐myogenic stem cells: HMGB1 recruits non–muscle‐derived stem cells such as MSCs and mesoangioblasts to reach the damaged areas and thus differentiate into muscle or replenish the muscle stem cell pool.

Muscle regeneration following damage or inflammation is a multifaceted process intricately linked to an augmented antioxidant response, with HMGB1 playing a pivotal role in shielding nascent muscle cells from oxidative stress induced by reactive oxygen species (ROS) [22]. Antioxidants act by scavenging ROS, thereby playing a protective role in ensuring cell proliferation and differentiation during the muscle regeneration process. Stem and precursor cell capacity in reaching, multiplying and fusing at the injury site is vital, with HMGB1 playing a pivotal role in these processes [66]. Oxidation eliminates muscle stem cell migration and differentiation into fibres in response to HMGB1 chemotaxis. Conversely, a reducing microenvironment promotes stem cell differentiation and muscle restoration. Neutralizing HMGB1 significantly suppresses LC3‐II formation, indicating an inhibition of autophagy, which subsequently leads to impaired angiogenesis. Administering recombinant HMGB1 promotes angiogenesis and improves muscle perfusion [35]. Fr‐HMGB1 can recruit stem cells and macrophages strongly associated with muscle atrophy. Fr‐HMGB1 exerts chemotaxis to recruit macrophages, which in turn undergo a series of reactions capable of transforming into the M2 phenotype, and Fr‐HMGB1 recruits stem cells to orchestrate tissue regeneration, all of which are undoubtedly disrupted by oxidation [7, 8, 9, 10, 22]. An oxidative environment in damaged muscle swiftly oxidizes HMGB1, compromising early injury repair and exacerbating inflammation. Conversely, in response to the oxidative damage caused by the original injury, a reducing microenvironment established by an effective antioxidant response can maintain the reducing activity of HMGB1, thereby prolonging its biological activity, reducing damage and promoting proliferation and repair [22]. Below we describe the possible effects of HMGB1 on muscle regeneration through four pathways (summarized in Figure 5).

### 4.1 HMGB1 and Vascular Regeneration

HMGB1 primarily induces skeletal muscle regeneration through its dual mechanisms: direct action on skeletal myoblasts, enhancing their recruitment to injured sites and facilitating angiogenesis [65]. HMGB1 can regulate angiogenesis by inducing human adipose‐derived stem cells (HASCs) via VEGF [67]. HMGB1 also promotes the homing of endothelial precursor cells to ischemic muscle, with RAGE acting as the principal receptor mediating this chemotactic activity [68]. It augments the expression of VEGF, stimulates the migration and proliferation of vascular smooth muscle cells, induces mesoangioblast migration and proliferation and supports angiogenesis. These actions contribute to the restoration or enhancement of the blood supply to injured tissues, such as muscle tissue, thereby facilitating the repair and regeneration processes [65, 69, 70]. The codelivery of VEGF and insulin‐like growth factor 1 (IGF1), which respectively promote angiogenesis and muscle regeneration, demonstrates a synergistic effect on muscle regeneration, with continuous delivery proving more effective than IGF1 alone. Satellite cells reside close to blood vessels [71], and their multiplication and differentiation are initiated simultaneously with the generation of new capillaries in regenerating muscle following injury [72]. Stimulating angiogenesis may increase the pool of myoblast stem cells available for muscle regeneration [73]. In addition to encouraging the generation of pro‐angiogenic cytokines from macrophages and ECs [74], HMGB1 also activates ECs, macrophages and mesoangioblasts, which contributes to angiogenesis [75].

### 4.2 HMGB1 and Stem Cell State

The administration of HMGB1 has been shown to accelerate the regeneration of muscle, bone and haematopoietic tissues in mice by inducing multiple stem cell types, including muscle stem cells, into the GAlert phase—a transitional state between G0 and G1. This transition allows the cells to promptly enter the cell cycle upon activation signals [76]. HMGB1 primarily facilitates the transition of human stem and progenitor cells into the GAlert phase through its fully reduced form [23]. Treatment with HMGB1 2 weeks prior to damage also expedited tissue regeneration, as HMGB1 promoted the corresponding stem cells to enter the GAlert state and maintain it until the injury stimulus, exerting a pro‐regenerative effect [23]. Lee et al. suggested that HMGB1 enhances the healing of various tissues by forming a heterogeneous complex with C–X–C motif chemokine ligand 12 (CXCL12), which then binds to C–X–C chemokine receptor 4 (CXCR4), facilitating the transition of quiescent stem cells to the GAlert phase [23].

### 4.3 HMGB1 and Myogenic Stem Cells

There are three overlapping but consecutive stages of muscle regeneration: the inflammatory reaction, the activation, differentiation and fusion of muscle satellite cells and the maturation and modification of newly generated myofibres [77]. Essential to this process are stem and precursor cells, with HMGB1 being crucial for their mobilization, proliferation and fusion [66]. Satellite cells, located between the sarcolemma and basal lamina, play a key role in muscle repair and growth. Myogenic factors drive their proliferation, differentiation and fusion to restore or create new muscle fibres [2, 77, 78]. In response to acute muscle injury or muscle wasting caused by conditions such as sepsis, cancer or muscular dystrophy, these cells, or myoblasts, differentiate to facilitate repair and regeneration [79, 80]. HMGB1 is crucial for skeletal muscle, as its deficiency impairs satellite cell differentiation, resulting in reduced levels of myogenin and IGF‐1 following injury [81]. RAGE expression in muscle is suppressed or downregulated at developmental completion and re‐expressed during certain pathological conditions. RAGE, which is influenced by HMGB1, promotes myogenesis by enhancing myogenic differentiation and myotube formation. HMGB1 acts upstream of RAGE to support myoblast differentiation, with its neutralization eliminating RAGE activation effects [82, 83]. Paired Box 7 (PAX7), found in both satellite and myogenic cells, delays myogenesis when its expression is maintained but is reduced in differentiated myoblasts [79]. In differentiated cells, the HMGB1/RAGE pathway stimulates the production of myogenin and suppresses Pax7, leading to reduced proliferation of myogenic cells and favouring symmetric division, which may consequently impede satellite cell self‐renewal but enhance myogenesis [79]. Notably, Sachdev et al. also found that HMGB1 and TLR4 participate in muscle protection and regeneration following muscle ischemia [84].

### 4.4 HMGB1 and Non‐Myogenic Stem Cells

Mesoangioblasts, vessel‐associated stem cells, can differentiate into various mesodermal cell types, such as skeletal muscle, smooth muscle and cardiac muscle, where the mesoangioblasts can be attracted to participate in the regeneration [85]. They cross the endothelium efficiently and migrate through tissue, guided by regenerating myofibres [86]. Intravenous administration of mesoangioblasts into dystrophic mice and dogs promotes muscle regeneration, evidenced by increased dystrophin expression, improved muscle morphology and function, enhanced contractility and restored locomotion [86, 87]. Further, mesoangioblasts can migrate into damaged muscle and do so more efficiently than other stem cells tested. Increased migration has been found to be correlated with increased muscle repair and motility [70]. Transplantation of mesoangioblasts also contributes to replenishing the muscle stem cell pool [88]. HMGB1 stimulates mesoangioblast proliferation and migration, acting as both a mitogenic and chemotactic factor. HMGB1 was shown to guide these cells to areas of the body where they are needed [69]. Mechanistically, HMGB1 activates the NF‐κB pathway via ERK, which regulates mesoangioblast migration and entry into damaged muscle [66, 70]. Polarized macrophages effectually evoked the migration of mesoangioblasts, which presupposed the secretion of HMGB1 by M1 cells and metalloproteinase‐9 (MMP‐9) by M2 cells [89]. Some studies have confirmed that HMGB1 can up‐regulate the expression of MMP‐9 [90].

HMGB1 also plays a significant role in influencing mesenchymal stem cells (MSCs), thereby exerting its effects on tissue regeneration. Researches have revealed that HMGB1 recruits MSCs through CXCL12 and activates their tissue repair mechanisms, increasing VEGF expression, promoting regeneration and enhancing cardiac function [91, 92]. It also stimulates the differentiation of MSCs into vascular cells, improving vascular injury [93]. Notably, MSCs, which are crucial participants in the healing of bone, cartilage, muscle, bone marrow stroma, tendon, fat and other connective tissues, can be drawn in from the locality and the bone marrow by the release of HMGB1 in injured tissues [24]. This allows MSCs to play a regenerative role in muscle tissue through HMGB1 chemotaxis. MSC therapy has been demonstrated to mitigate skeletal muscle atrophy by enhancing VEGF, which plays a role in angiogenesis and satellite cell pool maintenance, and balancing M1 and M2 macrophages [94].

## 5 Therapies Targeting HMGB1 for Muscle Atrophy

### 5.1 Box A on HMGB1

The structural makeup of HMGB1 includes two regions binding DNA, Box A and Box B, as well as an acidic C‐terminal. Box A can antagonize HMGB1 to neutralize its toxic effect by inhibiting activity and significantly reducing the level of HMGB1 [95]. Box A can yield the shrink in the release of HMGB1‐induced pro‐inflammatory TNF‐α and neutrophil recruitment to exert protective effects in LPS‐induced lung injury in mice [96]. Box A can also suppress the release of pro‐inflammatory TNF‐α and IL‐6, playing a protective role in ischemia–reperfusion injury in the heart [97]. The antitumour activity of Box A exerted in some tumour models has also been found [16]. Box A in the study recruited MSCs to reach the damaged myocardium and improved postinfarction remodelling [98]. The anti‐inflammatory properties of Box A can help mitigate inflammation associated with muscle atrophy. And it can induce stem cells to play a support role in regeneration.

### 5.2 Inhibition of HMGB1 by 1,25(OH)2D3

Vitamin D has been found to be involved in regulating the HMGB1 pathway and plays a role in muscle atrophy‐related diseases, including sepsis. Hemeoxygenase1 (HO‐1) was found to manipulate HMGB1 translocation and curb HMGB1 release, improving survival in sepsis model mice [99]. Rao et al. demonstrated that 1,25(OH)2D3 inhibits LPS‐induced HMGB1 secretion by targeting the nuclear factor erythroid 2‐related factor 2 (Nrf2)–HO1–HMGB1 pathway in macrophages [100]. Paricalcitol, a noncalcified vitamin D analogue, has been found to increase survival to sepsis and significantly reduce serum HMGB1 levels [100]. Similarly, the Nrf2–HO1–HMGB1 pathway has been shown to be effective at preventing myocardial ischemia and reperfusion injury [101]. Vitamin D insufficiency was found to be linked with decreased muscle strength, muscle atrophy, weakness and an increase in the risk of sarcopenia [102].

### 5.3 Inhibition of HMGB1 by miRNAs

MicroRNAs (miRNAs/MiR) are a highly conserved group of noncoding small RNAs controlling gene expression after transcription via degrading target mRNAs or restraining protein translation. They bind to the 3′‐UTR of mRNAs, leading to mRNA degradation or translational repression [103]. In sepsis, miRNAs play a crucial function in modulating the immunological response and release of inflammatory factors, and they can serve as potential biomarkers [104]. Studies have revealed that several miRNAs suppress the expression of HMGB1, being implicated in inflammatory reactions.

MiR‐212‐3p inhibited endotoxin‐induced inflammatory responses by directing HMGB1 to downregulate phosphorylation of p38MAPK and ERK [105]. Zhou et al. showed that miR‐205‐5b mimics lower HMGB1 levels in mouse macrophages while inhibiting miR‐205‐5b upregulates HMGB1 in sepsis [106]. MiR‐142‐3p was found to significantly inhibit HMGB1 3′‐UTR activity and suppress HMGB1 expression [107]. Investigators found activation of peroxisome proliferator‐activated receptor (PPAR)‐γ downregulated basal HMGB1 expression and attenuated LPS‐induced HMGB1 upregulation. PPAR‐δ and PPAR‐γ activation increases sirtuin 1 (SIRT1) expression, which regulates HMGB1 acetylation and inhibits LPS‐induced HMGB1 release, improving survival in endotoxemic models PPAR‐δ and PPAR‐γ activation increases SIRT1 expression, which regulates HMGB1 acetylation and inhibits LPS‐induced HMGB1 release, improving survival in endotoxemic models [107]. The deacetylase activity of SIRT1 is associated with the suppression of HMGB1 release [108]. MiR‐23a‐3p regulated LPS‐incurred inflammation by lowering the expression of HMGB1 [109]. MiRNAs‐23a was found to exert a function in muscle atrophy. Myotubes and muscle fibres, when forced to express miR‐23a, were protected against muscle atrophy [110]. In muscle cells, miR‐1192 directly inhibits HMGB1 mRNA translation through the microRNA binding site (miRBS), which is negated when HuR binds to its HuR binding site (HuRBS) [64].

### 5.4 Inhibition of HMGB1 by Curcumin

Curcumin, a polyphenolic compound in turmeric, exhibits anti‐inflammatory, antioxidant and anticancer properties. Delivering curcumin can preserve muscle mass and reduce muscle loss in several related models of animals and humans [111]. Curcumin downregulates muscle protein atrophy markers and signalling pathways, improving muscle atrophy and strength in cachectic and diabetic mice [112]. Curcumin enhances the body's muscle regeneration ability postinjury or unloading by directly promoting muscle precursor cell division and proliferation [113].

Curcumin can improve muscle atrophy by activating SIRT1. SIRT1‐mediated HMGB1 deacetylation inhibited its secretion and inflammation, significantly prolonging the survival time of septic mice [114]. Curcumin attenuates LPS‐induced muscle wasting by inhibiting muscle protein hydrolysis, downregulating the expression of Atrogin1 and MuRF‐1 and decreasing NF‐κB/p65 expression [115]. Notably, curcumin increases Nrf2 levels in muscle [111]. It was found that LPS‐triggered HMGB1 secretion could be reduced by activating the Nrf2–HO1 pathway [100].

### 5.5 Inhibition of HMGB1 by Glycyrrhizin

Glycyrrhizin is isolated from the roots and rhizomes of licorice and is one of the most essential active constituents. It effectively inhibits HMGB1 release and binds to HMGB1, reducing its binding capacity to DNA and suppressing inflammatory cascades by repressing chemokine and cytokine activities [9, 116]. Glycyrrhizin hinders HMGB1‐induced leukocyte chemotaxis post–muscle damage by disrupting the HMGB1–CXCL12 complex [9]. HMGB1 also exerts chemotaxis on muscle stem cells via CXCL12/CXCR4 [10], but it is still unknown whether glycyrrhizin will also inhibit the chemotaxis of reduced HMGB1 on muscle stem cells as it does on leukocyte chemotaxis. Glycyrrhizin has been found to inhibit HMGB1 and alleviate the effects of muscle atrophy. When directly bound to HMGB1, glycyrrhizin downregulates muscle atrophy‐related genes, Atrogin1 and MuRF‐1, and inhibits NF‐κB signalling, thereby improving muscle atrophy and weight loss [30].

### 5.6 Other Therapies for HMGB1 Inhibition

Inhibition of HMGB1 can also target its downstream receptors or pathways, such as soluble RAGE (sRAGE), RAGE, TLR4 and NF‐κB inhibition, which requires attention to the fact that these receptors have multiple upstream regulators and diverse functions. Alternative mRNA splicing and proteolytic cleavage of RAGE are the major pathways for the generation of sRAGE. It can act as a decoy receptor to intercept RAGE ligands, including HMGB1, and block their interaction with RAGE to exert inhibitory effects [117]. Modulating the activity of RAGE has therapeutic significance for skeletal muscles in certain disease conditions [117]. Inhibition of TLR4 ameliorated muscle atrophy by blocking HMGB1 signalling and preventing downstream receptor activation. [30]. Bay 11‐7082, an inhibitor of NF‐κB, has been demonstrated to lessen the loss of muscle or fat mass caused by sepsis [30, 118]. There are numerous other drugs that also inhibit HMGB1 in sepsis, which still need to be verified in related muscle atrophy studies. A detailed overview of these therapies is provided in Table S2.

### 5.7 Therapeutic Potential of HMGB1 in Regeneration

HMGB1 supports muscle repair and regeneration partially by facilitating angiogenesis [65, 69, 70, 73]. HMGB1 plays a crucial role in attracting stem cells and macrophages to the injury site, promoting tissue regeneration and accelerating the transition of stem cells from a quiescent to an active state through its reduced form [23, 76]. Research shows that HMGB1 is vital for muscle and vascular regeneration due to its promotive effects on muscle stem cells and mesoangioblasts. HMGB1 enhances the migration and proliferation of these cells, which are crucial for muscle repair. In satellite cells, HMGB1 supports their activation and differentiation, aiding in muscle restoration [79, 82, 83, 84]. For mesoangioblasts, HMGB1 promotes their movement to damaged muscles, improving regeneration and recovery [66, 69, 70, 86]. HMGB1 also recruits MSCs to differentiate into myoblasts.

During inflammation triggered by necrosis or injury, HMGB1 recruits inflammatory cells and causes local edema. In contrast, HMGB1 released by infiltrating leukocytes during regeneration orchestrates muscle repair and vascular remodelling, requiring an antioxidant response to maintain its reduced state [22, 81, 119]. Its reduced form, Fr‐HMGB1, has shown considerable research potential in muscle regeneration models due to its chemotactic, mobilizing and stem cell proliferative effects [7, 8, 9, 10]. However, it is susceptible to oxidative loss of these effects by environmental influences, so research into new antioxidant approaches is also promising. 3S‐HMGB1 is not affected by oxidation–reduction and has a promotional effect on muscle regeneration, thus providing a mechanism for sustained promotion of cell proliferation [10].

## 6 Conclusions and Perspectives

We provide a concise description of HMGB1's structure, function and modification, with an overview of how its redox state influences its function and downstream activities. Although current research has yet to fully elucidate HMGB1's exact role, it is evident that HMGB1 has a dual impact on muscle atrophy. HMGB1 participates in and induces muscle atrophy in sustained inflammatory conditions, while in its reduced form, it positively influences muscle regeneration. There is a close relationship between HMGB1 and major protein degradation pathways in muscle, such as the ubiquitin–proteasome and autophagy–lysosome systems, which are deeply linked and play a role in a number of disorders. However, further studies are needed to elucidate the role of HMGB1 and the interconnections therein in sepsis and related myopathies. This study also explores HMGB1's involvement in multiple forms of cell death associated with sepsis, including necroptosis, apoptosis, pyroptosis, ferroptosis, cuproptosis and NETosis. These septic cell deaths can exacerbate inflammation or affect myocytes, which can contribute to muscle atrophy. HMGB1 is closely associated with septic inflammation, attracting inflammatory cells, exacerbating inflammation, promoting cell death and activating downstream pathways, which are contributory to muscle atrophy. Understanding the relationship between HMGB1, these cell death processes, inflammation and their effects on muscle is crucial. We summarize existing pharmacological agents that inhibit HMGB1 or counteract its effects, noting that although some of these drugs have shown benefits in muscle‐related diseases, they lack validation in septic myopathy and muscle atrophy. This presents a promising avenue for future studies to evaluate whether inhibiting HMGB1 in sepsis could alleviate inflammation and muscle wasting. This review further explores HMGB1's role in muscle regeneration, emphasizing its potential to reduce muscle atrophy through various mechanisms. HMGB1 promotes angiogenesis, which facilitates the improvement of damaged muscles. It enhances stem cell potential, promotes stem cell proliferation towards muscle differentiation and replenishes the stem cell pool, all of which can help improve muscle atrophy. HMGB1 has a dual role in muscle, with each form exhibiting distinct functions. Therefore, comprehensive studies are needed to elucidate the detailed roles of Fr‐HMGB1, Ds‐HMGB1 and 3S‐HMGB1 in muscle atrophy and regeneration in sepsis.

In summary, the dual role of HMGB1 in muscle health is evident in its involvement in muscle wasting through the ubiquitination–proteasome system, autophagy–lysosome pathway, inflammatory response and programmed cell death and it also promotes muscle regeneration through vascular growth, enhanced stem cell potential and the differentiation of both myogenic and non‐myogenic stem cells. We highlight the need for comprehensive studies to clarify HMGB1's specific role in sepsis and muscle atrophy, focusing on ways to reduce inflammation and atrophy while promoting regeneration. We anticipate that insights into HMGB1 will guide future research, deepen the understanding of its mechanisms, drive the development of clinical therapies and enhance the feasibility of treating muscle atrophy.

## Conflicts of Interest

The authors declare no conflicts of interest.

## Supporting information


**Figure S1** HMGB1 and organ dysfunction in sepsis.
Table S1. Abbreviations.
Table S2. Drugs found to have an inhibitory effect on HMGB1 in sepsis.
