# Supplementary material for: HMGB1 in Septic Muscle Atrophy: Roles and Therapeutic Potential for Muscle Atrophy and Regeneration
Source: J Cachexia Sarcopenia Muscle. 2025 Feb 18;16(1):e13711. doi: 10.1002/jcsm.13711 (PMC11833301; doi:10.1002/jcsm.13711)
Supplement: Supplementary file 1 — Figure S1 HMGB1 and organ dysfunction in sepsis. Table S1. Abbreviations. Table S2. Drugs found to have an inhibitory effect on HMGB1 in sepsis. [file JCSM-16-e13711-s001.pdf]

SUPPLEMENTARY MATERIAL

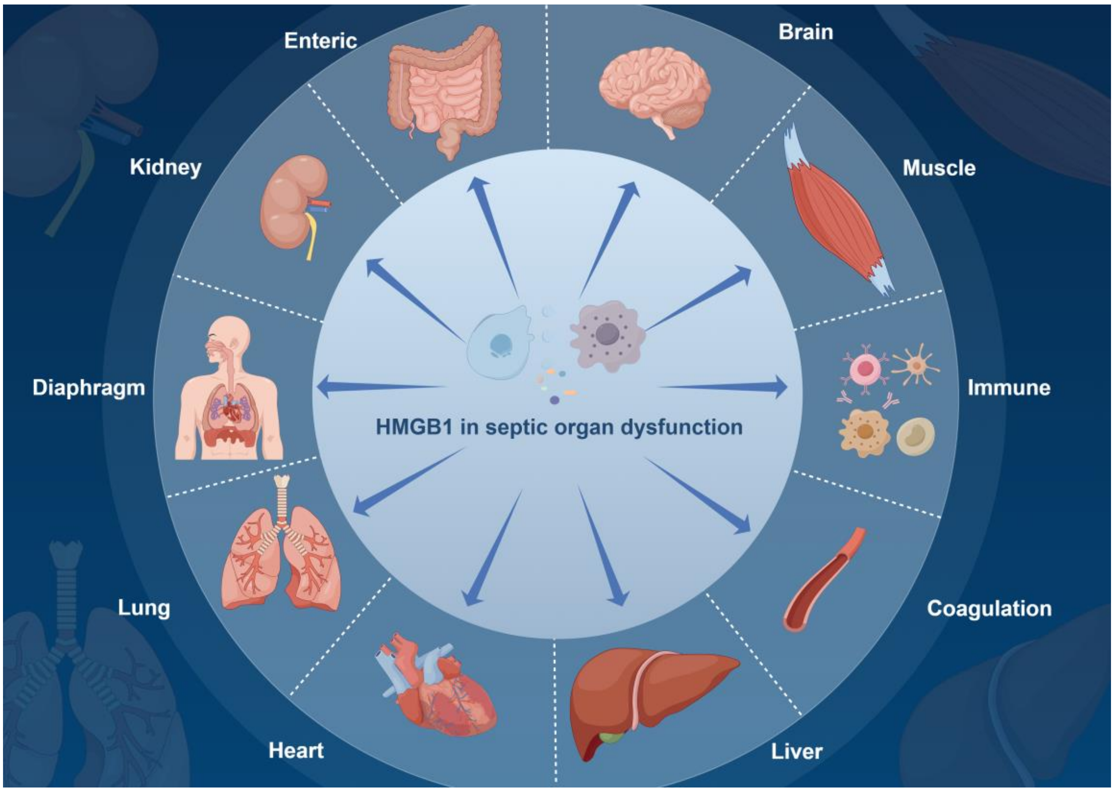

Figure S1. HMGB1 and organ dysfunction in sepsis. HMGB1 is a key mediator in the development of organ dysfunction during sepsis, contributing to: 1. Muscle Atrophy: Promotes muscle wasting through multiple pathways; 2. Immune Dysregulation: Causes imbalance in immune responses, resulting in hyperinflammation or immunosuppression; 3. Enteropathy: Leads to intestinal barrier dysfunction and systemic inflammation; 4. Diaphragmatic Dysfunction: Impairs diaphragmatic function, causing respiratory failure; 5. Pulmonary Injury: Contributes to acute lung injury and compromised gas exchange; 6. Myocardial Injury: Exacerbates myocardial inflammation and dysfunction; 7. Liver Injury: Induces hepatocellular damage and liver dysfunction; 8. Coagulopathy: Disrupts normal coagulation processes, leading to clotting disorders; 9. Sepsis-Associated Encephalopathy: Causes neuroinflammation and cognitive impairment; 10. Renal Injury: Contributes to acute kidney injury, impairing renal function.

Table S1. Abbreviations.

Abbreviations	Definition	Abbreviations	Definition
3S-HMGB1	Triple Serine-mutated HMGB1	MiRBS	MicroRNA Binding Site
AMPK	AMP-activated protein kinase	MMP-9	Metalloproteinase-9
ATP	Adenosine Triphosphate	MSCs	Mesenchymal Stem Cells
BCL-2	B-cell lymphoma 2	MuRF-1	Muscle-specific RING Finger Protein 1
DAPK	Death-associated protein kinase	MyHC	Myosin Heavy Chain
Ds-HMGB1	Disulfide HMGB1	NETosis	Neutrophil Extracellular Trap Formation
DTT	Dithiothreitol	NF-κB	Nuclear Factor Kappa-light-chain-enhancer of Activated B Cells
ECs	Endothelial Cells	Nrf2	Nuclear Factor Erythroid 2-Related Factor 2
ERK	Extracellular Signal-Regulated Kinase	Ox-HMGB1	Oxidized HMGB1
Fr-HMGB1	Fully Reduced HMGB1	PAX7	Paired Box 7
HASCs	Human Adipose-Derived Stem Cells	PKR	Double-stranded RNA-dependent Protein Kinase
HMGB1	High-Mobility Group Box 1	PPAR	Peroxisome Proliferator-Activated Receptor
HO-1	Hemeoxygenase 1	RAGE	Receptor for Advanced Glycation End-products
HuR	Human Antigen R	RIPK1	Receptor Interacting Protein Kinase 1
HuRBS	HuR Binding Site	ROS	Reactive Oxygen Species
IGF1	Insulin-like Growth Factor 1	S100B	S100 Calcium Binding Protein B
IL-1β	Interleukin-1 beta	sRAGE	Soluble RAGE
IL-6	Interleukin-6	SIRT1	Sirtuin 1
JAK	Janus Kinase	SQSTM1/p62	Sequestosome 1
LC3	Microtubule-associated Protein 1A/1B-Light Chain 3	STAT3	Signal Transducer and Activator of Transcription Protein 3
LPS	Lipopolysaccharide	TLR	Toll-like Receptor
MAPK	Mitogen-Activated Protein Kinase	TNF-α	Tumor Necrosis Factor-alpha
MiR	MicroRNAs	VEGF	Vascular Endothelial Growth Factor

Table S2. Drugs found to have an inhibitory effect on HMGB1 in sepsis.

Category	*General Mechanism of Action*	*Reference*
Drugs	Inhibit HMGB1 release or reduce HMGB1 levels	Paclitaxel[E1], Dabrafenib[E2], Biapenem[E3], Carbenoxolone[E4], GTS-21[E5], Metformin[E6], Ketamine[E7], Remifentanil[E8], Propofol[E9], Cilostazol[E10], Activated protein C[E11], D-Ala2-D-Leu5-enkephalin[E12], Magnesium sulfate[E13], Penehyclidine hydrochloride[E14], Reduning[E15], Cisplatin[E16]
	Inhibits the pro-inflammatory effects of HMGB1	Paclitaxel[E1], Dabrafenib[E2], Immunoglobulin[E17], Activated protein C[E11], Heparin[E18], Magnesium sulfate[E13], Reduning[E15]
	Binds to HMGB1 to exert inhibition	Heparin[E18]
Natural Compounds and Derivatives	Inhibit HMGB1 release or reduce HMGB1 levels	Curcumin[E19], Quercetin[E20], luteoloside[E15], Chloroquine[E21], Resveratrol[E22], Paeonol[E23], Emodin[E24], Fisetin[E25], Baicalein[E26], Sulforaphane[E27], Aloin[E28], Toddalolactone[E29], (-)-epigallocatechin-3-gallate[E30], Sesamin[E31], Persicarin[E32], Acteoside[E33], Indoprofen[E34], Angelica sinensis/Dang Gui [E35], tanshinone IIA sodium sulfonate[E36], Andrographolide[E37], rare ginsenosides[E38], Zingerone[E39], Rutin[E40], chlorogenic acid[E41], Forsythoside B[E42], Chromones and Flavanones [E43], Maslinic acid[E44], Mung Bean Coat Extract[E45], Decursin analogues[E46], protocatechuic aldehyde[E47], plumbagin[E48], Jujuboside B[E49], Tussilagone[E50], Glycyrrhizin[E51]
	Inhibits the pro-inflammatory effects of HMGB1	Quercetin[E20], luteoloside[E15], Chloroquine[E21], Sulforaphane[E27], (-)-epigallocatechin-3-gallate[E30], Indoprofen[E34], Calycosin[E52], Andrographolide[E37], rare ginsenosides[E38], Zingerone[E39], Rutin[E40], protocatechuic aldehyde[E47], Jujuboside B[E49] , Tussilagone[E50]
Bioactive Molecules	Inhibit HMGB1 release or reduce HMGB1 levels	MiRNA[E53], BoxA[E54], Histidine-rich glycoprotein[E55], specificity protein-1[E56], Stearoyl lysophosphatidylcholine[E57], Diketopiperazines[E58], cationic antibacterial polypeptide[E59], Cholinergic Agonists[E60]
	Inhibits the pro-inflammatory effects of HMGB1	MiRNAs[E53], BoxA[E54], Histidine-rich glycoprotein[E55], CRISPLD2[E61], Diketopiperazines[E58], cationic antibacterial polypeptide[E59]
	Binds to HMGB1 to exert inhibition	Haptoglobin β Protein[E62], Heparan Sulfate Octadecasaccharide (18-mer)[E63]
Chemical Reagents	Inhibit HMGB1 release or reduce HMGB1 levels	SGC-CBP30[E64], C-Methylcoumarinochromone[E65], PEGylated lysozymes[E66], Antagonism of Integrin CD11b[E67], THI-28[E68]
	Inhibits the pro-inflammatory effects of HMGB1	SGC-CBP30[E64], 4,4'-diphenylmethane-bis(methyl) carbamate (CM1)[E69], C-Methylcoumarinochromone[E65], PEGylated lysozymes[E66]
	Binds to HMGB1 to exert inhibition	FeTPPS[E70]

Additional References of Table S2

E1. Xu L, Hu G, Xing P, Zhou M, Wang D. Paclitaxel alleviates the sepsis-induced acute kidney injury via lnc-MALAT1/miR-370-3p/HMGB1 axis. Life Sci 2020; 262: 118505.

E2. Jung B, Kang H, Lee W, et al. Anti-septic effects of dabrafenib on HMGB1-mediated inflammatory responses. BMB Rep 2016; 49(4): 214-9.

E3. Kim J, Choo S, Sim H, Baek MC, Bae JS. Biapenem reduces sepsis mortality via barrier protective pathways against HMGB1-mediated septic responses. Pharmacol Rep 2021; 73(3): 786-95.

E4. Li W, Li J, Sama AE, Wang H. Carbenoxolone blocks endotoxin-induced protein kinase R (PKR) activation and high mobility group box 1 (HMGB1) release. Mol Med 2013; 19: 203-11.

E5. Hagiwara S, Iwasaka H, Hasegawa A, Asai N, Noguchi T. High-dose intravenous immunoglobulin G improves systemic inflammation in a rat model of CLP-induced sepsis. Intensive Care Med 2008; 34(10): 1812-9.

E6. Pavlov VA, Ochani M, Yang LH, et al. Selective alpha7-nicotinic acetylcholine receptor agonist GTS-21 improves survival in murine endotoxemia and severe sepsis. Crit Care Med 2007; 35(4): 1139-44.

E7. Tsoyi K, Jang HJ, Nizamutdinova IT, et al. Metformin inhibits HMGB1 release in LPS-treated RAW 264.7 cells and increases survival rate of endotoxaemic mice. Br J Pharmacol 2011; 162(7): 1498-508.

E8. Li K, Yang J, Han X. Ketamine attenuates sepsis-induced acute lung injury via regulation of HMGB1-RAGE pathways. Int Immunopharmacol 2016; 34: 114-28.

E9. Seo KH, Choi JW, Jung HS, Yoo H, Joo JD. The Effects of Remifentanil on Expression of High Mobility Group Box 1 in Septic Rats. Journal of Korean medical science 2017; 32(3): 542-51.

E10. Li S, Bao H, Han L, Liu L. Effects of propofol on early and late cytokines in lipopolysaccharide-induced septic shock in rats. J Biomed Res 2010; 24(5): 389-94.

E11. Chang KC. Cilostazol inhibits HMGB1 release in LPS-activated RAW 264.7 cells and increases the survival of septic mice. Thromb Res 2015; 136(2): 456-64.

E12. Bae JS, Rezaie AR. Activated protein C inhibits high mobility group box 1 signaling in endothelial cells. Blood 2011; 118(14): 3952-9.

E13. Li L, Ling Y, Huang M, et al. Heparin inhibits the inflammatory response induced by LPS and HMGB1 by blocking the binding of HMGB1 to the surface of macrophages. Cytokine 2015; 72(1): 36-42.

E14. Tang CW, Feng WM, Du HM, Bao Y, Zhu M. Delayed administration of D-Ala2-D-Leu5-enkephalin, a delta-opioid receptor agonist, improves survival in a rat model of sepsis. Tohoku J Exp Med 2011; 224(1): 69-76.

E15. Jiang J, Chen Q, Chen X, Li J, Li S, Yang B. Magnesium sulfate ameliorates sepsis-induced diaphragm dysfunction in rats via inhibiting HMGB1/TLR4/NF-kappaB pathway. Neuroreport 2020; 31(12): 902-8.

E16. Yang Q, Liu X, Yao Z, Mao S, Wei Q, Chang Y. Penehyclidine hydrochloride inhibits the release of high-mobility group box 1 in lipopolysaccharide-activated RAW264.7 cells and cecal ligation and puncture-induced septic mice. J Surg Res 2014; 186(1): 310-7.

E17. Wang Z, Chen W, Li Y, et al. Reduning injection and its effective constituent luteoloside

protect against sepsis partly via inhibition of HMGB1/TLR4/NF-kappaB/MAPKs signaling pathways. J Ethnopharmacol 2021; 270: 113783.
E18. Pan P, Cardinal J, Dhupar R, et al. Low-dose cisplatin administration in murine cecal ligation and puncture prevents the systemic release of HMGB1 and attenuates lethality. J Leukoc Biol 2009; 86(3): 625-32.
E19. Karimi A, Pourreza S, Vajdi M, et al. Evaluating the effects of curcumin nanomicelles on clinical outcome and cellular immune responses in critically ill sepsis patients: A randomized, double-blind, and placebo-controlled trial. Frontiers in nutrition 2022; 9: 1037861.
E20. Tang D, Kang R, Xiao W, et al. Quercetin prevents LPS-induced high-mobility group box 1 release and proinflammatory function. Am J Respir Cell Mol Biol 2009; 41(6): 651-60.
E21. Yang M, Cao L, Xie M, et al. Chloroquine inhibits HMGB1 inflammatory signaling and protects mice from lethal sepsis. Biochem Pharmacol 2013; 86(3): 410-8.
E22. Yang Y, Li S, Yang Q, et al. Resveratrol reduces the proinflammatory effects and lipopolysaccharide- induced expression of HMGB1 and TLR4 in RAW264.7 cells. Cell Physiol Biochem 2014; 33(5): 1283-92.
E23. Miao J, Zhong J, Lan J, et al. Paeonol attenuates inflammation by confining HMGB1 to the nucleus. J Cell Mol Med 2021; 25(6): 2885-99.
E24. Liu FJ, Gu TJ, Wei DY. Emodin alleviates sepsis-mediated lung injury via inhibition and reduction of NF-kB and HMGB1 pathways mediated by SIRT1. The Kaohsiung journal of medical sciences 2022; 38(3): 253-60.
E25. Yoo H, Ku SK, Han MS, Kim KM, Bae JS. Anti-septic effects of fisetin in vitro and in vivo. Inflammation 2014; 37(5): 1560-74.
E26. Chen HM, Liou SF, Hsu JH, et al. Baicalein inhibits HMGB1 release and MMP-2/-9 expression in lipopolysaccharide-induced cardiac hypertrophy. Am J Chin Med 2014; 42(4): 785-97.
E27. Lee I-C, Kim DY, Bae J-S. Sulforaphane Reduces HMGB1-Mediated Septic Responses and Improves Survival Rate in Septic Mice. The American Journal of Chinese Medicine 2017; 45(06): 1253-71.
E28. Yang S, Lee W, Lee BS, et al. Aloin Reduces HMGB1-Mediated Septic Responses and Improves Survival in Septic Mice by Activation of the SIRT1 and PI3K/Nrf2/HO-1 Signaling Axis. Am J Chin Med 2019; 47(3): 613-33.
E29. Ni J, Zhao Y, Su J, et al. Toddalolactone Protects Lipopolysaccharide-Induced Sepsis and Attenuates Lipopolysaccharide-Induced Inflammatory Response by Modulating HMGB1-NF-kappaB Translocation. Front Pharmacol 2020; 11: 109.
E30. Li W, Ashok M, Li J, Yang H, Sama AE, Wang H. A major ingredient of green tea rescues mice from lethal sepsis partly by inhibiting HMGB1. PLoS One 2007; 2(11): e1153.
E31. Li ZL, Gao M, Yang MS, Xiao XF, Liu JJ, Yang BC. Sesamin attenuates intestinal injury in sepsis via the HMGB1/TLR4/IL-33 signalling pathway. Pharm Biol 2020; 58(1): 898-904.
E32. Kim TH, Ku SK, Bae JS. Persicarin is anti-inflammatory mediator against HMGB1-induced inflammatory responses in HUVECs and in CLP-induced sepsis mice. J Cell Physiol 2013; 228(4): 696-703.
E33. Seo ES, Oh BK, Pak JH, et al. Acteoside improves survival in cecal ligation and puncture-induced septic mice via blocking of high mobility group box 1 release. Molecules and cells 2013; 35(4): 348-54.

E34. Bi X, Yan X, Jiang B, et al. Indoprofen exerts a potent therapeutic effect against sepsis by alleviating high mobility group box 1-mediated inflammatory responses. Toxicol Appl Pharmacol 2021; 433: 115778.
E35. Wang H, Li W, Li J, et al. The aqueous extract of a popular herbal nutrient supplement, Angelica sinensis, protects mice against lethal endotoxemia and sepsis. The Journal of nutrition 2006; 136(2): 360-5.
E36. Li W, Li J, Ashok M, et al. A cardiovascular drug rescues mice from lethal sepsis by selectively attenuating a late-acting proinflammatory mediator, high mobility group box 1. J Immunol 2007; 178(6): 3856-64.
E37. Chen G, Hou Y, Li X, Pan R, Zhao D. Sepsis-induced acute lung injury in young rats is relieved by calycosin through inactivating the HMGB1/MyD88/NF-kappaB pathway and NLRP3 inflammasome. Int Immunopharmacol 2021; 96: 107623.
E38. Lee W, Ku S, Yoo H, Song K, Bae J. Andrographolide inhibits HMGB1-induced inflammatory responses in human umbilical vein endothelial cells and in murine polymicrobial sepsis. Acta Physiol (Oxf) 2014; 211(1): 176-87.
E39. Kim JE, Lee W, Yang S, et al. Suppressive effects of rare ginsenosides, Rk1 and Rg5, on HMGB1-mediated septic responses. Food Chem Toxicol 2019; 124: 45-53.
E40. Lee W, Ku SK, Bae JS. Zingerone reduces HMGB1-mediated septic responses and improves survival in septic mice. Toxicol Appl Pharmacol 2017; 329: 202-11.
E41. Yoo H, Ku SK, Baek YD, Bae JS. Anti-inflammatory effects of rutin on HMGB1-induced inflammatory responses in vitro and in vivo. Inflamm Res 2014; 63(3): 197-206.
E42. Lee CH, Yoon SJ, Lee SM. Chlorogenic acid attenuates high mobility group box 1 (HMGB1) and enhances host defense mechanisms in murine sepsis. Mol Med 2013; 18(1): 1437-48.
E43. Jiang W-L, Yong X, Zhang S-P, Zhu H-B, Jian H. Forsythoside B Protects Against Experimental Sepsis by Modulating Inflammatory Factors. Phytotherapy Research 2012; 26(7): 981-7.
E44. Tuan NQ, Lee W, Oh J, et al. Flavanones and Chromones from Salicornia herbacea Mitigate Septic Lethality via Restoration of Vascular Barrier Integrity. J Agric Food Chem 2015; 63(46): 10121-30.
E45. Lee W, Lee H, Lee T, Park EK, Bae JS. Inhibitory functions of maslinic acid, a natural triterpene, on HMGB1-mediated septic responses. Phytomedicine 2020; 69: 153200.
E46. Zhu S, Li W, Li J, Jundoria A, Sama AE, Wang H. It Is Not Just Folklore: The Aqueous Extract of Mung Bean Coat Is Protective against Sepsis. Evidence-based complementary and alternative medicine : eCAM 2012; 2012: 498467.
E47. Lee W, Yuseok O, Lee C, et al. Suppressive activities of KC1-3 on HMGB1-mediated septic responses. Biochem Pharmacol 2019; 163: 260-8.
E48. Xu Y, Jiang WL, Zhang SP, Zhu HB, Hou J. Protocatechuic aldehyde protects against experimental sepsis in vitro and in vivo. Basic Clin Pharmacol Toxicol 2012; 110(4): 384-9.
E49. Zhang Z, Deng W, Kang R, et al. Plumbagin Protects Mice from Lethal Sepsis by Modulating Immunometabolism Upstream of PKM2. Mol Med 2016; 22: 162-72.
E50. Kim N, Kim C, Ryu SH, Bae JS. Jujuboside B Inhibited High Mobility Group Box Protein 1-Mediated Severe Inflammatory Responses in Human Endothelial Cells and Mice. Journal of medicinal food 2023; 26(1): 40-8.
E51. Kim YK, Yeo MG, Oh BK, et al. Tussilagone Inhibits the Inflammatory Response and

Improves Survival in CLP-Induced Septic Mice. Int J Mol Sci 2017; 18(12).
E52. Gu J, Ran X, Deng J, et al. Glycyrrhizin alleviates sepsis-induced acute respiratory distress syndrome via suppressing of HMGB1/TLR9 pathways and neutrophils extracellular traps formation. Int Immunopharmacol 2022; 108: 108730.
E53. Chen W, Ma X, Zhang P, Li Q, Liang X, Liu J. MiR-212-3p inhibits LPS-induced inflammatory response through targeting HMGB1 in murine macrophages. Exp Cell Res 2017; 350(2): 318-26.
E54. Gong Q, Xu JF, Yin H, Liu SF, Duan LH, Bian ZL. Protective effect of antagonist of high-mobility group box 1 on lipopolysaccharide-induced acute lung injury in mice. Scand J Immunol 2009; 69(1): 29-35.
E55. Gao S, Wake H, Sakaguchi M, et al. Histidine-Rich Glycoprotein Inhibits High-Mobility Group Box-1-Mediated Pathways in Vascular Endothelial Cells through CLEC-1A. iScience 2020; 23(6): 101180.
E56. Yang H, Wang H, Wang Y, et al. The haptoglobin beta subunit sequesters HMGB1 toxicity in sterile and infectious inflammation. Journal of internal medicine 2017; 282(1): 76-93.
E57. Liu ZM, Wang X, Li CX, et al. SP1 Promotes HDAC4 Expression and Inhibits HMGB1 Expression to Reduce Intestinal Barrier Dysfunction, Oxidative Stress, and Inflammatory Response after Sepsis. J Innate Immun 2022; 14(4): 366-79.
E58. Quan H, Bae HB, Hur YH, et al. Stearoyl lysophosphatidylcholine inhibits LPS-induced extracellular release of HMGB1 through the G2A/calcium/CaMKKbeta/AMPK pathway. Eur J Pharmacol 2019; 852: 125-33.
E59. Arnold K, Xu Y, Sparkenbaugh EM, et al. Design of anti-inflammatory heparan sulfate to protect against acetaminophen-induced acute liver failure. Sci Transl Med 2020; 12(535).
E60. Zhang S, Pei L, Qu J, et al. CRISPLD2 attenuates pro-inflammatory cytokines production in HMGB1-stimulated monocytes and septic mice. American journal of translational research 2021; 13(5): 4080-91.
E61. Lee W, Ku SK, Park S, Kim KM, Choi H, Bae JS. Inhibitory Effect of Three Diketopiperazines from Marine-Derived Bacteria on HMGB1-Induced Septic Responses in Vitro and in Vivo. Am J Chin Med 2016; 44(6): 1145-66.
E62. Shibusawa K, Murakami T, Yomogida S, Tamura H, Nagaoka I. Antimicrobial cathelicidin peptide CAP11 suppresses HMGB1 release from lipopolysaccharide-stimulated mononuclear phagocytes via the prevention of necrotic cell death. Int J Mol Med 2009; 23(3): 341-6.
E63. Wang H, Liao H, Ochani M, et al. Cholinergic agonists inhibit HMGB1 release and improve survival in experimental sepsis. Nat Med 2004; 10(11): 1216-21.
E64. Wang X, Li Z, Bai Y, et al. A small molecule binding HMGB1 inhibits caspase-11-mediated lethality in sepsis. Cell Death Dis 2021; 12(4): 402.
E65. Bi X, Jiang B, Zhou J, et al. CBP Bromodomain Inhibition Rescues Mice From Lethal Sepsis Through Blocking HMGB1-Mediated Inflammatory Responses. Front Immunol 2020; 11: 625542.
E66. Feng L, Zhu M, Zhang M, et al. Amelioration of compound 4,4'-diphenylmethane-bis(methyl)carbamate on high mobility group box1-mediated inflammation and oxidant stress responses in human umbilical vein endothelial cells via RAGE/ERK1/2/NF-κB pathway. Int Immunopharmacol 2013; 15(2): 206-16.
E67. Lee W, Lee D, Lee Y, et al. Isolation, Synthesis, and Antisepsis Effects of a C-Methylcoumarinochromone Isolated from Abronia nana Cell Culture. J Nat Prod 2018; 81(5):

1173-82.
E68. Lee W, Park EJ, Kwak S, Kim Y, Na DH, Bae JS. PEGylated lysozymes with anti-septic effects in human endothelial cells and in mice. Biochemical and biophysical research communications 2015; 459(4): 662-7.
E69. Zhou H, Li Y, Gui H, et al. Antagonism of Integrin CD11b Affords Protection against Endotoxin Shock and Polymicrobial Sepsis via Attenuation of HMGB1 Nucleocytoplasmic Translocation and Extracellular Release. J Immunol 2018; 200(5): 1771-80.
E70. Kim HS, Park EJ, Park SW, Kim HJ, Chang KC. A tetrahydroisoquinoline alkaloid THI-28 reduces LPS-induced HMGB1 and diminishes organ injury in septic mice through p38 and PI3K/Nrf2/HO-1 signals. Int Immunopharmacol 2013; 17(3): 684-92.
